# Macrophage sensing of the acidic milieu: BRD4 condensates in focus

**DOI:** 10.1093/lifemeta/loaf035

**Published:** 2025-09-16

**Authors:** Dan Zhang, Vini Tiwari, Christopher K Glass

**Affiliations:** Department of Cellular and Molecular Medicine, University of California San Diego, La Jolla, CA 92093, United States; Department of Cellular and Molecular Medicine, University of California San Diego, La Jolla, CA 92093, United States; Department of Cellular and Molecular Medicine, University of California San Diego, La Jolla, CA 92093, United States; Department of Medicine, University of California San Diego, La Jolla, CA 92093, United States

**Wu *et al.* uncover bromodomain-containing protein 4 (BRD4) condensates as direct intracellular pH sensors that reprogram macrophage inflammatory gene expression. This discovery reveals how acidic microenvironments reshape immunity and opens new questions about chromatin-based control of inflammation.** 

The idea of inflammation has always carried imagery of fire—the term itself is derived from the Latin inflammatio, “to set on fire”. This metaphor reflects its double-edged nature: while inflammation is essential for defending against infection and repairing injury, it can just as easily harm the body’s own tissues when unresolved. Normally, acute inflammation subsides once the threat is cleared, but when the resolution fails, chronic inflammation arises as a central driver of chronic diseases, ranging from autoimmune disorders to cancer [1, 2]. One striking yet often overlooked feature of severe or persistent inflammation is the acidification of the local tissue microenvironment, arising from hypoxia, altered metabolism, and the accumulation of lactate and other metabolites [3]. Macrophages, as central orchestrators of innate and adaptive immunity, are uniquely positioned at the interface of these environmental changes. They respond to pathogen-derived signals as well as sterile tissue injury, integrating multiple cues to calibrate inflammatory programs. Yet, the molecular mechanisms by which macrophages sense and adapt to a lowered pH milieu, and how this process influences the quality of inflammatory responses, have remained poorly defined. In a recent study published in *Cell*, Wu *et al.* uncover a novel mechanism by which macrophages sense intracellular pH and reprogram gene transcription through biomolecular condensates, identifying bromodomain-containing protein 4 (BRD4) as a direct and selective pH sensor [4].

Wu *et al.* reveal an unexpected role for extracellular acidification as a determinant of macrophage gene programs. Upon lipopolysaccharide (LPS)-induced Toll-like receptor (TLR) activation, classical primary response genes such as chemokine (C-X-C motif) ligand 1 (*Cxcl1*), tumor necrosis factor (*Tnf*), and nuclear factor-kappa-B inhibitor alpha (*Nfkbia*) remained largely unaffected by acidic pH (approximately 6.5). In contrast, a subset of secondary inflammatory cytokines (interleukin-1beta (*Il1b*), *Il6*, and *Il12b*) were strongly repressed, while genes including interferon beta-1 (*Ifnb1*), tumor necrosis factor superfamily member 9 (*Tnfsf9*), and adrenomedullin (*Adm*) were paradoxically upregulated. These findings suggest that macrophages do not simply attenuate their activation under acidic conditions but rather integrate pH as a signaling input to qualitatively remodel transcriptional outputs. Transcriptomic profiling coupled with linear deconvolution modeling further classified macrophage genes into three categories: pH-insensitive (innate immune pathways), pH-repressed (cytokine signaling and adaptive immunity), and pH-synergistic (co-activated by TLR signaling and acidity, e.g., *Ifnb1* and *Il23a*). This framework highlights that innate responses are largely resilient to acidity, whereas adaptive and interferon programs are preferentially tuned by pH, revealing a previously unrecognized layer of immune regulation.

Mechanistically, the authors demonstrate that this pH-dependent transcriptional control cannot be attributed to canonical pH sensors (G-protein coupled receptors GPR65 and GPR68), hypoxia-responsive HIF factors, or inflammasome activation. Nor is it explained by defects in translation, replication, or canonical TLR4 and interferon signaling. Acidic pH does not induce global chromatin compaction but instead exerts gene-specific effects: enhancer activity is selectively disrupted, transcriptional kinetics are altered, and trimethylation of histone H3 lysine 4 (H3K4me3) levels is reduced across pH-antagonized loci. Strikingly, interferon-β secretion and activation of signal transducer and activator of ­transcription 1 (STAT1) are enhanced under acidic conditions, underscoring that pH functions not as a general suppressor of inflammation but as a selective modulator that reshapes the ­transcriptional landscape.

**Figure 1:**
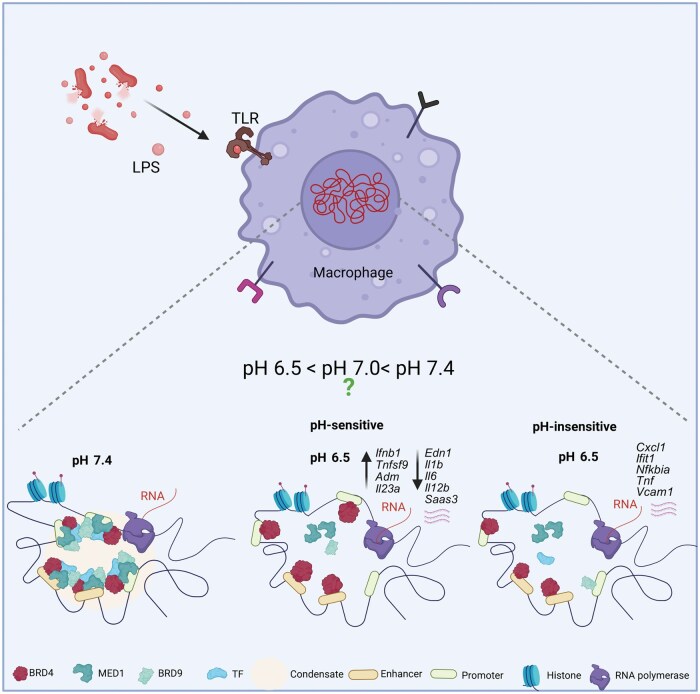
From acidic milieu to chromatin regulation: contextualizing BRD4 condensate dissolution in macrophages. Macrophage sensing of bacterial LPS results in reduced intracellular pH. Wu *et al.* demonstrate *in vitro* that this causes BRD4 condensates to dissolve, resulting in selective alterations in gene expression, inhibiting genes dependent on BRD4 condensates while enabling activation of genes that drive acute inflammatory response. Unresolved questions include whether small pH shifts within the physiological range (pH 7.4 → approximately 7.0 → ≤ 6.4) are sufficient to alter BRD4 condensate stability, enhancer function, and transcriptional feedback *in vivo*, and the extent to which this mechanism is used in other cell types and pathophysiologic contexts. Figure created in Biorender.com.

A major highlight of the study is the identification of BRD4 as a molecular pH sensor. BRD4, a bromodomain and extraterminal domain (BET) family chromatin reader, binds acetylated histones via its bromodomains and promotes RNA polymerase II elongation through recruitment of the positive transcription elongation factor b (p-TEFb). By occupying enhancers and super-enhancers, it scaffolds transcriptional complexes to regulate cell identity and stimulus-responsive gene programs. Wu *et al.* show that BRD4 forms liquid-like transcriptional condensates via its intrinsically disordered regions (IDRs), and that these condensates are exquisitely sensitive to intracellular acidification. At pH 6.5, BRD4–mediator complex subunit 1 (MED1) condensates dissolve, disrupting enhancer–promoter communication mediated by BRD4–MED1 and BRD4–BRD9 interactions. This dissolution selectively impairs pH-sensitive inflammatory genes while sparing housekeeping and early TLR-induced genes (Fig. 1). Histidine clusters within BRD4 IDRs confer this sensitivity: mutating histidines renders condensates acid-resistant, while restoring neutral pH reinstates condensates and reactivates suppressed genes. Importantly, BRD4 not only senses pH but also regulates glycolysis and intracellular pH via transcriptional programs, acting as a homeostatic feedback controller of inflammation. These findings provide direct evidence that histidine-rich IDRs can act as biophysical pH sensors, linking microenvironmental acidity to transcriptional reprogramming.

Together, this study identifies intracellular pH as a previously unrecognized regulatory cue that qualitatively reprograms macrophage inflammatory responses. By connecting BRD4 condensate dynamics with pH sensing, the study bridges immunometabolism, chromatin biology, and phase separation. The demonstration that macrophages deploy distinct gene programs under acidic conditions has broad implications, from chronic inflammatory diseases to tumor immunity, where acidity is a hallmark of the microenvironment. These findings raise the possibility that targeting pH-sensitive condensates may offer new therapeutic opportunities to counteract immune suppression in acidic tissues.

This work also opens several important avenues for future investigation. While Wu *et al.* focused on LPS-stimulated bone-marrow-derived macrophages (BMDMs) to dissect how pH reshapes inflammatory responses, it remains unclear whether this regulatory logic extends to other ligand-induced pathways or to sterile inflammation triggered by tissue damage. Not all inflammatory contexts generate acidic microenvironments—for example, thioglycolate-induced sterile peritonitis engages the triggering receptor expressed on myeloid cells 2 (TREM2)-dependent signaling pathway without overt acidification. Understanding how macrophages propagate or restrain inflammation in non-acidic settings will be critical for understanding the full scope of pH sensing. A key question is whether BRD4 condensate-mediated, pH-dependent enhancer regulation also operates in tissue-resident macrophages, and whether such mechanisms exhibit tissue-specific differences. Since BRD4 is broadly expressed, pH-sensitive condensate dissolution in non-immune cells may also communicate metabolic stress signals to macrophages, extending this regulatory axis beyond the immune compartment [7]. Establishing the physiological relevance of this mechanism *in vivo* will be crucial for understanding its role in human disease and may ultimately guide strategies to modulate inflammation therapeutically. Sex differences in response to inflammation add another layer of complexity and vary strongly across tissues. An open question is whether BRD4 condensate dissolution during pH sensing contributes to sex-specific chromatin and signaling differences, and thereby contributes to sexually dimorphic inflammatory outcomes [8].

More broadly, the concept that phase-separated condensates act as dynamic sensors of environmental cues is still in its infancy. Although *in vitro* systems have provided valuable insights into condensate assembly and dissolution, it remains a major challenge to determine how these structures function within the complexity of living tissues. Condensate-like assemblies have been implicated in neurodegeneration, cancer, and chronic inflammation [9], but direct evidence linking them to gene regulation and immune function *in vivo* is limited. The study by Wu *et al.* provides a foundation to ask whether similar pH-sensitive condensates exist in other immune lineages, and whether their dysregulation contributes to disease-associated states such as tumor immunosuppression or neuroinflammation. Addressing these questions will require integrated strategies that combine biochemical, genetic, and imaging approaches with *in vivo* disease models, including quantitative live-tissue imaging of condensate dynamics and single-cell multi-omics and proteomics to define condensate composition and enhancer connectivity under inflammatory stress.

An open question related to the extent to which the findings of Wu *et al.* will apply to physiologic and pathophysiologic settings is the point at which changes in pH result in dissolution of BRD4 condensates. Wu *et al.* only examined two discrete pH conditions (7.4 and 6.5). While pH 7.4 is consistent with that of blood, most tissues under homeostatic conditions are closer to pH 7. Small deviations within this physiological range may be particularly important, since tissue pH can dip transiently to 6.5 or lower in select contexts, such as skeletal muscle during extreme exercise or in segments of the kidney that regulate acid–base balance [5, 6]. A critical question, therefore, is at what threshold intracellular acidification is sufficient to dissolve BRD4 condensates. If dissolution only occurs at or below about 6.5, it may represent a relatively rare event in acute inflammation, metabolic acidosis, or tissue-specific niches. If, instead, it occurs closer to neutral pH, this mechanism could have far broader relevance across multiple physiological and pathological settings.

Finally, the temporal dimension of pH sensing by BRD4 condensates raises new possibilities. In acute inflammation, such as LPS challenge, transient BRD4 condensate dissolution restrains secondary cytokines and provides an adaptive brake. Low pH dissolves BRD4 condensates, functionally inhibiting its activity at pH-sensitive genes. By contrast, studies with BET inhibitors show that pharmacological BRD4 inhibition can induce replication stress, R-loops, and activation of the cyclic GMP-AMP synthase–stimulator of interferon genes (cGAS–STING) in other contexts [10, 11, 12]. Whether prolonged, pH-driven condensate dissolution could produce similar DNA damage-linked innate immune responses remains an open question. This possibility is especially relevant in chronic conditions such as aging, where persistent low-grade tissue acidification develops through cumulative metabolic dysfunction, hypoxia, and mitochondrial decline—the hallmarks of “inflammaging” [13]. Ultimately, exploring these temporal and contextual dimensions may reveal not only new principles of gene regulation but also therapeutic opportunities to manipulate condensate dynamics in pathologically acidic microenvironments.

This study broadens our understanding of how the immune system senses and integrates environmental cues to calibrate inflammatory responses. By uncovering intracellular pH and BRD4 condensates as critical regulators, Wu *et al.* pave the way for deeper exploration of how biophysical properties of tissues intersect with gene regulation and immune responses. Establishing the physiological relevance of this mechanism *in vivo* will be crucial for understanding its role in human disease and may ultimately guide strategies to therapeutically modulate inflammation.
